# *Plasmodium falciparum* Mutant Haplotype Infection during Pregnancy Associated with Reduced Birthweight, Tanzania

**DOI:** 10.3201/eid1909.130133

**Published:** 2013-09

**Authors:** Daniel T. R. Minja, Christentze Schmiegelow, Bruno Mmbando, Stéphanie Boström, Mayke Oesterholt, Pamela Magistrado, Caroline Pehrson, Davis John, Ali Salanti, Adrian J.F. Luty, Martha Lemnge, Thor Theander, John Lusingu, Michael Alifrangis

**Affiliations:** National Institute for Medical Research, Tanga Centre, Tanzania (D.T.R. Minja, B. Mmbando, P. Magistrado, M. Lemnge, J. Lusingu);; University of Copenhagen, Copenhagen, Denmark (D.T.R. Minja, C. Schmiegelow, P. Magistrado, C. Pehrson, A. Salanti, T. Theander, J. Lusingu, M. Alifrangis);; Wenner-Gren Institute, Stockholm, Sweden (S. Boström);; Radboud University Nijmegen Medical Centre, Nijmegen, the Netherlands (M. Oesterholt, A.J.F. Luty);; Kilimanjaro Christian Medical Centre, Moshi, Tanzania (D. John)

**Keywords:** Plasmodium falciparum, malaria, mutations, haplotype, pregnancy, drug resistance, polymorphisms, dihydrofolate reductase, dihydropteroate synthetase, Tanzania, intermittent preventive treatment, sulfadoxine-pyrimethamine, sextuple, low birth weight, parasites

## Abstract

Intermittent preventive treatment during pregnancy with sulfadoxine–pyrimethamine (IPTp-SP) is a key strategy in the control of pregnancy-associated malaria. However, this strategy is compromised by widespread drug resistance from single-nucleotide polymorphisms in the *Plasmodium falciparum* dihydrofolate reductase and dihydropteroate synthetase genes. During September 2008–October 2010, we monitored a cohort of 924 pregnant women in an area of Tanzania with declining malaria transmission. *P. falciparum* parasites were genotyped, and the effect of infecting haplotypes on birthweight was assessed. Of the genotyped parasites, 9.3%, 46.3%, and 44.4% had quadruple or less, quintuple, and sextuple mutated haplotypes, respectively. Mutant haplotypes were unrelated to SP doses. Compared with infections with the less-mutated haplotypes, infections with the sextuple haplotype mutation were associated with lower (359 g) birthweights. Continued use of the suboptimal IPTp-SP regimen should be reevaluated, and alternative strategies (e.g., intermittent screening and treatment or intermittent treatment with safe and effective alternative drugs) should be evaluated.

Pregnancy-associated malaria is a leading cause of maternal anemia and low birthweight (*1*). Measures to prevent pregnancy-associated malaria include insecticide-treated nets, treatment with effective antimalarial drugs, and administration of intermittent preventive treatment during pregnancy with sulfadoxine–pyrimethamine (IPTp-SP) (*2*). IPTp-SP is given at least twice during pregnancy, with doses 1 month apart (*3*,*4*). Studies have shown that IPTp-SP reduces the incidence of anemia, clinical malaria, low birthweight, and parasite prevalence at delivery (*3*,*5*–*9*).

SP acts by inhibiting the *Plasmodium falciparum* dihydropteroate synthetase and dihydrofolate reductase enzymes, respectively (*10*,*11*). However, resistance to the combined drug (SP) is widespread among the *P. falciparum* population in sub-Saharan Africa; this resistance is caused by accumulation of point mutations in the *P. falciparum* dihydropteroate synthetase *(Pfdhf*r*)* and dihydrofolate reductase *(Pfdhp*s*)* genes (*12*,*13*). An increased number of point mutations in these genes is associated with augmented resistance to SP in vivo (*14*). There is sufficient evidence to support that the triple *Pfdhfr* mutation asparagine 51 to isoleucine (N51I), cysteine 59 to arginine (C59R), and serine 108 to asparagine (S108N) in combination with double *Pfdhps* mutant alanine 437 to glycine (A437G) and lysine 540 to glutamic acid (K540E)—forming quintuple mutant haplotypes—confer a high risk for treatment failure in malaria-infected children and nonpregnant adults who receive SP treatment (*14*). In addition, recent reports have shown that an increase in *Pfdhps* mutations at alanine 581 to glycine (A581G), further escalating the risk for even higher levels of resistance (*15*).

Because *P. falciparum* parasite resistance to SP is high, most likely because of the high prevalence of quintuple mutant haplotypes, use of the drug to treat uncomplicated malaria has been abandoned in many parts of eastern Africa. In Tanzania, SP was replaced in 2006 by artemether-lumefantrine for the management of uncomplicated malaria (*4*). The high prevalence (>50%) of the K540E mutation, which is found almost exclusively as the quintuple mutant haplotype, has also resulted in poor SP efficacy when used as intermittent preventive treatment in infants (*16*). However, other studies have indicated that IPTp-SP is still efficacious in some areas with high resistance (*17*). Nonetheless, with increased *P. falciparum* resistance, the usefulness of IPTp-SP might be compromised (*18**–**20*).

It has not been known whether there is an association between *P. falciparum* sextuple mutant haplotypes and poor pregnancy outcome. To determine if there is a relationship, we conducted a prospective cohort study in northeastern Tanzania in an area with declining malaria transmission (*21*). The study received ethical approval from the Tanzania Medical Research Coordinating Committee (reference no. NIMR/HQ/R.8a/Vol. IX/688). All procedures were conducted in accordance with the Declaration of Helsinki and Good Clinical and Laboratory Practices. All participants gave written informed consent.

## Methods

### Study Design and Samples

The study was conducted during September 2008–October 2010 in Korogwe District in the Tanga Region of northeastern Tanzania, where *P. falciparum* is the predominant malaria-causing species. A prospective cohort of 924 pregnant women was monitored from first attendance at the antenatal clinic through delivery. The study has been described in detail (*22*–*24*). In brief, pregnant women with a gestational age of <24 weeks, as estimated by using ultrasound, were enrolled if they had lived in Korogwe District for >6 months and were willing to give birth at Korogwe District Hospital. Study participants attended 3 additional prescheduled antenatal visits at weeks 26, 30, and 36 of pregnancy, and they were attended to by a study nurse/clinician. Obstetric history and maternal anthropometric measurements were recorded for all women (*23*). A venous blood sample was collected at each antenatal clinic visit, and venous and placental blood samples were collected at the time of delivery. 

### Diagnosis, Treatment, and Prevention of Malaria

All blood samples were tested for malaria parasites by using a rapid diagnostic test (RDT) (Parascreen, Zephyr Biomedicals, Goa, India; Paracheck Pf, Orchid Biomedical Systems, Mumbai, India; or ParaHIT-f, Span Diagnostics Ltd, Surat, India) and by microscopy. Blood smears for women with negative RDT results were examined retrospectively, whereas those for women with positive RDT results were examined immediately if deemed necessary by the study physician for a treatment decision (*25*). Parasite density was determined as the number of asexual stage parasites/200 leukocytes (500 leukocytes if <10 parasites) and converted to the number per microliter, as described (*22**,**25*); >100 fields were double-examined before a blood smear was declared negative.

Women with positive RDT results were treated with the antimalarial drug artemether-lumefantrine (Coartem Dispersible, Norvatis Corporation, Suffern, New York, USA) or with quinine. For infections occurring during the first trimester of pregnancy, quinine sulfate–coated tablets (ELYS Chemical Industries Ltd, Nairobi, Kenya) were used, and for severe cases, quinine dihydrochloride injection (Vital Healthcare PVT Ltd, Mumbai, India) was used.

Two doses of IPTp-SP (Sulphadar, Shelys Pharmaceutical Ltd, Dar es Salaam, Tanzania) were given >1 month apart as directly observed treatment; each dose contained 1,500 mg of sulfadoxine and 75 mg of pyrimethamine. Women with a gestational age of >20 weeks at enrollment were given the first IPTp-SP dose at the study-inclusion visit and the second dose during the third trimester. Women with a gestational age of <20 weeks at enrollment were given the first dose at 20 weeks of gestation. Women who had received IPTp-SP before study inclusion but earlier than recommended by the World Health Organization (i.e., after quickening in the second trimester) received a second dose after 20 weeks of gestation, and a third dose was given in the third trimester. Use of SP from private pharmacies/drug shops for malaria treatment before and after study inclusion was also documented. All study participants were provided with a voucher for procuring insecticide-treated nets.

### Laboratory Methods and Birthweight Measurements 

EDTA-preserved venous blood was used to estimate hemoglobin levels (KX-21N Automated Hematology Analyzer, Sysmex, Kobe, Japan). Live newborns whose birthweights were measured by using a spring scale (Fazzini, Vimodrone, Italy) with an accuracy of <50 g or a digital strain gauge scale (ADE, Hamburg, Germany) with an accuracy of <10 g within 24 h of delivery were included in the birthweight analysis. Newborns with severe malformations, twins, and those born to women with preeclampsia were excluded from analyses because these conditions can severely affect birthweight (*23*).

EDTA-preserved blood (50 μL) was spotted on Whatman number 3 filter paper (VWR– Bie & Berntsen, Herlev, Denmark), dried at room temperature, and stored in separate zip-lock bags. DNA was extracted by using the Chelex 100 method, as described (*26*). The DNA supernatant was transferred to a 96-well PCR plate and stored at −20°C until use. The parasite DNA was amplified by outer and nested *P. falciparum*–specific PCRs, as described (*27*); the products were analyzed by electrophoresis in 1.5% ethidium bromide–stained gel, as described (*22*).

To determine the multiplicity of infections, block 2 of the merozoite surface protein 2 domain was amplified by using fluorescent PCR (*28*). The results were analyzed by using GeneScan software, version 3.7 (Applied Biosystems, Naerum, Denmark).

Parasite DNA was amplified by outer and nested PCR with specific primers targeting the *Pfdhfr* and *Pfdhps* genes, as described (*29*). Single-nucleotide polymorphisms (SNPs) in the *Pfdhfr* and *Pfdhps* genes were identified by using a sequence-specific oligonucleotide probe ELISA technique, as described (*29*) with minor modifications. In brief, we used sequence-specific oligonucleotide probes targeting *Pfdhfr* codons c50/51 CI/CN, c59 (C/R), c108 (S/N/T), and c164 (I/L) and *Pfdhps* codons c436/437 (AA/AG/SA/SG/FG), c540 (K/E), c581 (A/G), and c613 (A/S). Individual SNPs were combined to deduce the different infecting mutant haplotypes.

### Data Management and Statistical Analyses

We double-entered and validated data in Microsoft Access version 2007 (Redmond, WA, USA). Statistical analyses were conducted by using Stata version 10 (StataCorp, College Station, TX, USA) deploying parametric and nonparametric methods, as appropriate. The effect of infecting allelic haplotypes on birthweights was investigated by using multiple linear regression and dichotomized (as 6 and <6 SNPs) to infecting haplotypes; variables with a p<0.20 in univariate analysis were included in the multivariate models. By using a stepwise backward elimination approach, we obtained final models including only variables with a p<0.10. A 2-sided p-value of <0.05 was considered significant. Final models included only women without missing values.

## Results

### Demographic and Parasitologic Characteristics of the Study Cohort

Of 1,171 screened pregnant women, 995 met the study inclusion criteria; 924/995 women completed follow-up. For the entire study cohort, 5,555 venous and placental blood samples were collected during antenatal care and at delivery. Among the women completing follow-up, 76 had a total of 96 episodes of malaria. Some women had >1 infection, as determined by RDT and/or blood smears. The median asexual parasite density for study participants was 2,570 asexual stages/μL (range 40–390,749).

A total of 91 samples from women with RDT parasite-positive results were examined by *P. falciparum*–specific PCR, and 65 (71.4%) were positive. Of these 65 samples, 54 (83.1%) from 49 women were successfully typed in subsequent molecular analyses; 21 (38.9%) of these 54 samples were obtained at study enrollment. The median age of women with samples included in the genotyping analysis was 22 years (range 17−35), and at study inclusion, they had a median gestational age of 17.7 weeks (range 6.9–23.9). Overall, 91.8% of the women in the study cohort received 2 doses of IPTp-SP.

### Multiplicity of Infections and *P. falciparum dhfr*/*dhps* Genotypes

Sixty percent of the genotyped parasites were polyclonal, with a mean of 2.5 clones/sample (range 1.0–8.0 clones/sample). Of the few women with repeat *P. falciparum* infections and sequence-specific oligonucleotide probe ELISA data (n = 5), 1 had a recrudescent and 1 had a new infection; genotype results of the 3 remaining infections were inconclusive.

**Figure 1:**
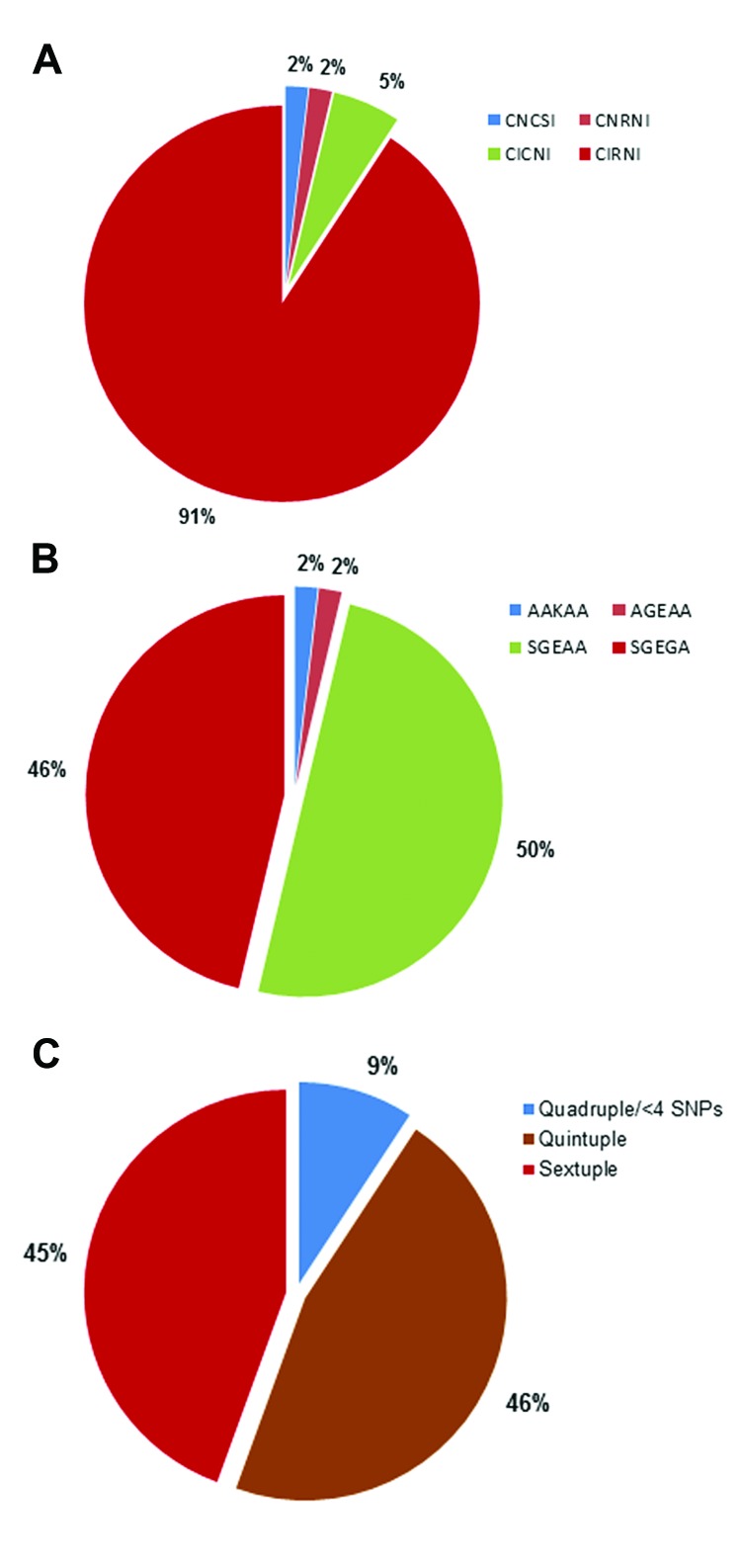
*Pfdhfr/Pfdhps* haplotyping results for *Plasmodium falciparum* parasite isolates from 54 parasite isolates in 49 pregnant women, Korogwe District, Tanga Region, Tanzania, September 2008–October 2010. A) Proportion of single nucleotide polymorphisms (SNPs) conferring sulfadoxine–pyrimethamine resistance on the *P. falciparum* dihydrofolate reductase gene (*Pfdhfr*) at codons C50, N51I, C59R, S108N, and L164I, resulting in allelic haplotypes CNCSI (wild-type), CICNI (double *Pfdhfr* mutant), CNRNI (double *Pfdhfr* mutant), and CIRNI (triple *Pfdhfr* mutant). B) Proportion of SNPs conferring sulfadoxine–pyrimethamine resistance on the *P. falciparum* dihydropteroate synthetase (*Pfdh*p*s*) gene at codons S436A, A437G, K540E, A613S/T, and A581G with allelic haplotypes AKAA (wild-type), AGEAA (double *Pfdhps* mutant), SGEAA (double *Pfdhps* mutant), and SGEGA (triple *Pfdhps* mutant). C) Proportions of *Pfdhfr/Pfdhps* quadruple or less, quintuple, and sextuple mutant haplotypes from the cohort of pregnant women. The derivations of the allelic haplotypes were based on a combination of 2 or 3 *Pfdhfr* SNPs with 2 or 3 *Pfdhps* SNPs forming quadruple (4 SNPs), quintuple (5 SNPs), and sextuple (6 SNPs) haplotypes. Quadruple haplotype or less included 4 SNPs (quadruple) and triple *Pfdhfr* (CIRNI, n = 1) or double *dhps* (SGEAA, n = 1) with wild-type *dhfr* (CNCSI, n = 1) or wild-type *dhps* (AKAA, n = 1). Less than 4 haplotypes had 1 triple or double mutation in 1 gene combined with a wild-type mutation in the other gene.

Of the 54 parasite isolates, 49 (90.7%) were triple *Pfdhfr* mutants (CIRNI [C50, 51I, 59R, 108N, and I164]); 4 (7.4%) isolates were double *Pfdhfr* mutants CICNI/CNRNI; and the remaining 1 (1.9%) isolates was wild-type CNCSI (Figure 1A). Two major *Pfdhps* haplotypes were identified in the 54 isolates: the double mutant AGEAA/SGEAA (A436/S436, A437G, and K540E) haplotype in 28 (51.9%) isolates and the triple mutant *Pfdhps* SGEGA (S436A, A437G, K540E, and A581G) haplotype in 25 (46.3%) isolates (Figure 1B). An AAKAA wild-type haplotype was identified in only 1 (1.9%) of the 54 isolates (Figure 1B).

The different SNPs were combined to generate the following mutant *Pfdhfr*/*Pfdhps* haplotypes: quadruple haplotype (double *Pfdhfr* [CICNI/CNRNI] with double *Pfdhps* [AGEAA/SGEAA]); quintuple haplotypes (triple *Pfdhfr* with double *Pfdhps* [CNRNI–AGEAA/SGEAA] or double *Pfdhfr* with triple *Pfdhps* [CICNI–SGEGA]); and sextuple haplotype (triple *Pfdhfr* [CIRNI] and triple *Pfdhps* [SGEGA]). The only *Pfdhfr* CNCSI wild-type isolate was a double *Pfdhps* (SGEAA) mutant, and the only *Pfdhps* AAKAA wild-type haplotype was a triple *Pfdhfr* (CIRNI) mutant. The isolates with these 2 haplotypes were grouped as quadruple or less when generating the combined *Pfdhfr/Pfdhps* haplotypes. The quadruple or less, quintuple, and sextuple haplotypes were observed for 9.3%, 46.3%, and 44.4% of the malaria infections, respectively (Figure 1, panel C). For the woman with repeated infections, the highest level of mutation was used in the analyses on pregnancy outcome.

### Trends of *Pfdhfr* and *Pfdhps* Allelic Haplotypes by Gestational Age

**Figure 2:**
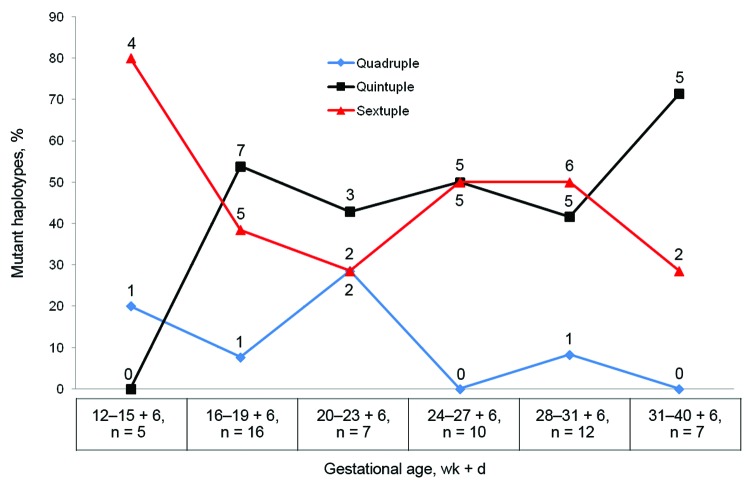
Proportion of mutant *Plasmodium falciparum* dihydrofolate reductase and dihydropteroate synthetase haplotypes among pregnant women, Korogwe District, Tanga Region, Tanzania, September 2008–October 2010. Proportions are shown by gestational age; partial weeks are indicated by the number of days. Numbers above and below data points are the number of mutant haplotypes; total numbers (n) are shown below the graph.

Quadruple and less mutated haplotypes were observed mainly during early pregnancy, but the quintuple and sextuple haplotypes were observed throughout pregnancy (Figure 2); there was no clear trend indicating a selection by IPTp-SP use for the most mutated haplotypes (Table 1). Among the women who did not receive IPTp-SP before malaria infection, 3 with quintuple and 1 with sextuple haplotype infections reported using SP for treatment of suspected malaria before study inclusion. Even when both exposures to SP (i.e., as IPTp-SP and as SP treatment) were taken into account, no trend toward accumulation of resistant haplotypes as a result of SP use was observed (Table 1).

**Table 1:** Stratification of *Plasmodium falciparum* mutant haplotypes among pregnant women, by exposure of the women to SP before infection, Korogwe District, Tanga Region, Tanzania, September 2008–October 2010*

Participant exposure to SP	No. (%) women with infecting allelic haplotype		
	Quadruple or less, n = 5	Quintuple, n = 25	Sextuple, n = 24
Did not receive IPTp–SP (n = 28)	4 (14.3)	12 (42.9)	12 (42.9)
Received first dose of IPTp–SP (n = 18)	1 (5.6)	8 (44.4)	9 (50.0)
Received second dose of IPTp–SP (n = 8)	0	5 (62.5)	3 (37.5)
Had previous exposure to SP (n = 30)†	1 (3.3)	16 (53.0)	13 (43.0)

*SP, sulfadoxine–pyrimethamine; IPTp–SP, intermittent preventive treatment during pregnancy with SP. †Received IPTp-SP or SP used as treatment for malaria before study inclusion.

### Effect of Sextuple Haplotypes on Pregnancy Outcome

There were no statistically significant differences in the characteristics of women infected with highly mutated parasites and those with less mutated parasites with respect to age, gravidity, and anthropometry (Table 2). Of the 26 women with quintuple or less haplotype infections and 23 with sextuple haplotype infections, 24 and 20, respectively, received 2 doses of IPTp-SP. Two women with quintuple or less haplotype infections and 2 with sextuple haplotype infections received no or 1 dose of IPTp-SP, and 1 woman with sextuple haplotype infection received 3 doses. The levels of parasitemia were also similar between the 2 groups, and there were no differences in the incidence of fever (axillary temperature >37.5°C) or level of hemoglobin (g/dL) at the time of infection or at delivery (Table 2).

**Table 2:** Demographic and biological characteristics for 49 pregnant women infected with *Plasmodium falciparum* sextuple or less mutant haplotypes, Korogwe District, Tanga Region, Tanzania, September 2008–October 2010*

Characteristic	*P. falciparum* mutant allelic haplotype								p value‡
	Less than sextuple				Sextuple				
	No.†	Median†	Range or %		No.†	Median†	Range or %		
Maternal age, y	26.0	22.5	17.0–35.0		23.0	21.0	17.0–32.0		0.38
Gravidity	26				23				0.81
Primi-/secundigravidae	20		77%		17		74%		
Multigravidae (3–8 pregnancies)	6		23%		6.0		26%		
Gestational age, wk									
At study inclusion	26.0	17.6	6.8–23.8		23.0	16.3	8.3–22.3		0.54
At delivery	26.0	39.7	32–43.1		22.0	40.0	35.1–42.6		0.82
Owned bed net	26	19	73%		23	11	48%		0.07
MUAC									
At study inclusion, cm	26	26	20–32		23	24	22.0–37.0		0.07
At delivery, cm	23.0	25.5	21.4–31.6		20.0	25.0	21.2–30.0		0.22
Child’s birthweight, g§	19	3,148 ± 434¶			17	2,822 ± 436¶			**0.03**
Children with birthweight <2,500 g	19	1	5%		17	2	12%		0.82
*z*-score at delivery	18.0	−0.11 ± 1.27¶			17.0	−0.88 ± 1.07¶			0.06
SGA at delivery	19	2	11%		17	6	35%		0.11
Weight of placenta, g	17	645	381–780		15.0	492	307–800		0.16
Hemoglobin level, g/dL									
At delivery#	16.0	11.2 ± 1.8¶			13.0	11.1 ± 1.8¶			0.80
At time of infection	25.0	10.0 ± 1.4¶			22.0	10.3 ± 1.8¶			0.56
Fever at time of infection	25	1	4%		23	2	8.7%		0.60**
Parasitemia, IE/μL††	17	2,565	42–10,1208		18	1,895	40–390,749		0.64
>1 infection‡‡	26	1	4%		23	7	30%		**0.02**
>2 doses of IPTp	26	24	92%		23	21	91%		0.90

*MUAC, mid upper arm circumference; SGA, small gestational age; IE, infected erythrocytes; IPTp, intermittent preventive treatment during pregnancy.  †Data are no. or median no. unless otherwise indicated in column one or by ¶. ‡Unless otherwise indicated, all medians were compared by using the Mann Whitney Rank sum test, means were compared by using the Student *t*-test, and proportions were compared by using the χ^2^ test. **Bold** font indicates statistical significance (p<0.05). §Among the 49 study participants, 1 had preeclampsia, 2 delivered twins, and 1 delivered a newborn with severe malformation. The birthweight of 39 newborns (including 1 pair of twins and the child born with severe malformation) was measured within 24 h of birth. Only the 36 singleton newborns without malformation were included in the analyses.  ¶Data are mean ± SD. #Hemoglobin levels were measured for many women after delivery; however, only levels measured before delivery were included in the analyses. Low hemoglobin after delivery might be due to ante- and postpartum bleeding rather than antenatal events (e.g., malaria infection).  **Fisher exact test. ††Parasitemia is only stated for 17 nonsextuple and 18 sextuple infections because some infections were rapid diagnostic test–positive but blood smear–negative. Sequence-specific oligonucleotide probe ELISA on filter paper was, however, still possible despite the very low level of parasitemia.  ‡‡No. infections is based on all infections detected in the woman by using a rapid diagnostic test and/or blood smear, regardless of whether sequence-specific oligonucleotide probe ELISA was conducted.

Among the 49 newborns born to women for whom *P. falciparum* strains were genotyped, there was 1 case of preeclampsia, 2 twin deliveries, and 1 newborn with congenital malformation. Among the remaining 45 singleton newborns, 36 had their birthweight measured within 24 hours of delivery and, thus, were eligible for analysis. There was no difference in the distribution of haplotypes between mothers of the included and excluded newborns (data not shown).

The median birthweight for newborns of women with sextuple haplotype infections was significantly lower than that for newborns of women with less mutated haplotype infections (Table 2). When using *z*-scores (adjusting for gestational age at delivery and sex of newborn) based on a Tanzanian reference chart from the same cohort (*23*), borderline significance toward lower *z*-scores in women with sextuple mutant haplotype infections was observed (p = 0.06). There was no difference in the incidence of low birthweight newborns or of newborns that were small for gestational age at birth among women with sextuple haplotype and less mutated haplotype infections (Table 2). When the effect on birthweight was further analyzed by using a stepwise multiple linear regression model adjusting for covariates, newborns of women with sextuple mutant haplotype infections had a significantly reduced birthweight (mean reduction 359 g; 95% CI, −601 to −117) compared with newborns of women with quintuple or less haplotype infections (p = 0.005) (Table 3) Covariates for the linear regression model were mid upper arm circumference at study inclusion, parasite density, hemoglobin level, number of IPTp-SP doses, gestational age at inclusion and at delivery, number of infections, maternal age, weight/weight gain during pregnancy, HIV status, place of delivery, sex of the newborn, and gravidity.

**Table 3:** Determinants of birthweight for children born to 49 pregnant women with *Plasmodium falciparum* genotype data, Korogwe District, Tanga Region, Tanzania, September 2008–October 2010*

Variable	Univariate analysis				Multivariate analysis†		
	Coefficient	95% CI	p value		Coefficient	95% CI	p value
MUAC at study inclusion, cm	49	1 to 97	0.047		15	**−**28 to 59	0.47
Gestational age, wk							
At study inclusion	**−**20	**−**63 to 22	0.340				
At delivery	90	**−**23 to 204	0.120		90	1 to 179	**0.048**
Sex of newborn, F	**−**106	**−**432 to 221	0.520				
Maternal age, y	37	10 to 64	0.009		2	**−**30 to 35	0.890
Gravidity (>2 pregnancies)	477	166 to 788	0.004		434	152 to 716	**0.004**
Maternal weight at study inclusion, kg	9	**−**12 to 30	0.370				
Maternal weight gain, kg							
From study inclusion to ANV3	38	**−**47 to 124	0.372				
From ANV3–ANV4	**−**22	−72 to 28	0.370				
Infecting haplotype**,** sextuple	**−**326	**−**621 to **−**31	0.031		**−**359	**−**601 to **−**117	**0.005**
Parasite density, per 1,000 IE/μL	0.09	−3 to 3	0.95				
No. infections‡	**−**308	**−**649 to 34	0.08		**−**143	**−**426 to 140	0.31
No. IPTp doses before delivery	**−**303	**−**614 to 8	0.06		**−**231	**−**498 to 35	0.087
Maternal HIV status positive	230	**−**308, to 769	0.390				
Place of delivery							
Hospital	**−**86	**−**381 to 209	0.56				
Other than hospital	**−**12	**−**696 to 671	0.971				

*Effect on birthweight in the multivariate analyses is stated for all variables with p<0.2 in the univariate analyses. In the final model, only variables with p<0.10 were included in the model. MUAC, mid upper arm circumference; ANV3, antenatal clinic visit at gestational week 30; ANV3–4, ANV at gestational week 30–36; IE, infected erythrocytes. †Blank spaces indicate no data/information. **Boldface** indicates statistical significance (p<0.05). ‡No. infections is based on all infections detected in the woman by using a rapid diagnostic test and/or blood smear, regardless of whether sequence specific oligonucleotide probes–ELISA was conducted.

Women in whom sextuple haplotype parasites were identified more often had repeated infections than those in whom less mutated haplotypes were identified (Table 2). In the univariate analysis, there was a borderline significance toward decreased birthweight after repeated infections (p = 0.08). However, this effect was not observed in the multivariate model (Table 3).

There was a trend toward decreased birthweight with increasing numbers of IPTp-SP doses received (Table 3). This trend was explained by the fact that newborns of the 3 women who received 0 or 1 IPTp-SP dose had much higher birthweights than newborns of the 33 women who received 2 doses (3,690 g vs. 2,920 g, respectively; p = 0.04). However, in the entire cohort (924 women) there was no association between the number of IPTp-SP doses and birthweight (data not shown). Furthermore, the number of IPTp-SP doses received before time of infection and the time between the most recent IPTp-SP dose and when infection occurred was not associated with birthweight. Adding these variables to the multivariate model did not alter the result (data not shown).

## Discussion

IPTp-SP is still being used in areas where prevalence of *Pfdhfr/Pfdhps* mutations is high (*17*), although widespread resistance is likely to affect the protective effect of this strategy in preventing pregnancy-associated malaria. The continued use of this suboptimal drug for IPTp might promote further development of resistance because resistant strains are likely to have a fitness advantage.

This study showed that despite administration of a full IPTp-SP course to most of the study participants, the women were not fully protected, and malaria infections occurred in 8.2% (76/924) of the women. Point mutations in *Pfdhfr* and/or *Pfdhps* genes were observed in all genotyped parasites. Quadruple or less mutated haplotypes were mainly observed early during pregnancy, whereas quintuple and sextuple mutated haplotypes were encountered throughout pregnancy. Harrington et al. (*20*) indicated that IPTp-SP use was associated with increased prevalence of parasites with mutations at codon A581G. However, we did not observe an association between exposure to SP before infection and increased A581G mutant parasite prevalence (Table 1). This lack of association in this study could be due to the fact that the parasite populations were already largely saturated with the highly mutated strains.

Of concern, the high prevalence of the *Pfdhfr/Pfdhps* sextuple haplotype was associated with reduced birthweight. Previous studies have reported an association between mutation level and adverse pregnancy outcomes (*18**–**20*) rather than, as we show, an adverse effect on birthweight specifically associated with sextuple haplotype parasites. For the entire cohort of 924 women, a negative association between malaria and fetal growth/birthweight has been reported (*25*).

We converted birthweights to *z*-scores by using a reference chart of birthweights for infants born to healthy women in the 924-person study cohort (*23*). By using the *z*-scores, we standardized the difference between individual birthweight and the mean population birthweight adjusted for sex of newborn and gestational age at delivery. The disadvantage of this conversion is that any inherent uncertainty in the reference chart is imputed into the birthweight measure. This could explain why the difference in *z*-scores between the sextuple and less mutated haplotypes was only borderline significant (Table 2). The multivariate model on the effect of infection with the sextuple haplotype on birthweight was already adjusted for gestational age at delivery, and the results were not altered when sex of the newborn was included (data not shown).

Harrington et al. (*18*) observed that the use of IPTp-SP increased parasite growth. We did not find a substantial difference in parasite density between the different haplotypes, nor did we find that the time between IPTp-SP use and infection altered the effect of the haplotype on birthweight. Therefore, IPTp-SP does not seem to increase parasite density, and differences in parasite density cannot explain the observed effect of the sextuple haplotype on birthweight. The presence of highly mutated *P. falciparum* genotypes that cannot be cleared by IPTp-SP exposes women to persistent and chronic malaria infections. In our study, sextuple haplotype parasite infections were associated with reduced weight of the placenta, although this difference did not reach statistical significance. Low placental weight indicates poor development of the placenta, which has been associated with pregnancy-associated malaria (*30**, **31*). In vitro assays with extravillous trophoblasts showed that serum and plasma from *P. falciparum*–infected pregnant women inhibited extravillous trophoblast invasion and migration, offering a possible explanation for the pathophysiologic events that may cause impaired placentation, reduced placental weight, and low infant birthweight (*32*). Therefore, longer lasting infections, altering placental development among the women with sextuple haplotype infections, could explain the reduced birthweights observed in our study.

Harrington et al. (*18*) reported that SP in itself could have detrimental effects on the health of newborns. Our findings do not indicate that SP in itself causes lower birthweight. We did find a borderline significant association between the number of IPTp-SP doses and reduced birthweight among the 36 newborns; however, this association was not seen when we evaluated the effect of IPTp-SP doses on birthweight in the entire cohort of 924 women (data not shown).

Despite regular screening, using RDTs, for malaria among pregnant women and treatment with effective antimalarial drugs, it is worrisome that birthweights for newborns of women infected with sextuple mutated parasites were lower than those for newborns of women infected with less mutated parasites. This finding underscores the need to evaluate the effect of these mutations among populations using IPTp-SP at a wider scale.

Because of the changing epidemiology of malaria, which has transformed the transmission pattern from high to low in large parts of sub-Saharan Africa (*21**,**33*), including the study area, it is likely that many pregnant women, irrespective of gravidity, will have little immunity against pregnancy-associated malaria. The decreasing prevalence of malaria and the fact that isolates with sextuple mutations had a significant effect on birthweight underscores the question of whether IPTp-SP should be continued or replaced by intermittent screening and treatment and/or with an alternative drug for IPTp. SP might be teratogenic in the first trimester of pregnancy (*34*), and most other currently available antimalarial drugs are either teratogenic or their efficacy and safety profiles among pregnant women are still poorly known (*35*); thus, there is an urgent need to conduct further efficacy and safety studies to determine alternative drugs for IPTp. Until then, we suggest that screening by RDTs during pregnancy may allow early case detection and prompt treatment with effective antimalarial drugs (*22*).

This study demonstrates that sextuple *Pfdhfr*/*Pfdhps* mutated haplotypes are prevalent in the study area and that these highly SP-resistant parasites are associated with a significant reduction in birthweight of newborns of malaria-infected women. Presumably, the presence of these highly mutated *P. falciparum* genotypes is largely unaffected by IPTp-SP and expose women to persistent and chronic malaria infection, and this effect, rather than SP by itself, has detrimental effects on the health of newborns. We observed highly mutated haplotypes even before IPTp-SP was used and throughout pregnancy, indicating saturation of the population with resistant parasites. Therefore, continued use of the suboptimal IPTp-SP regimen should urgently be reevaluated, and its replacement with screening and treatment or with an alternative safe and effective antimalarial drug for IPTp should be considered.
